# Proteomic and Phosphoproteomic Characteristics of Multiple Brain Regions in Rhesus Macaques After SARS‐CoV‐2 Infection

**DOI:** 10.1002/mco2.70906

**Published:** 2026-08-06

**Authors:** Qiaochu Wang, Yanan Zhou, Tao Ding, Yan Sun, Yehong Yang, Yue Wu, Jiangfeng Liu, Shuaiyao Lu, Juntao Yang

**Affiliations:** ^1^ State Key Laboratory of Common Mechanism Research for Major Diseases, Department of Biochemistry and Molecular Biology, Institute of Basic Medical Sciences Chinese Academy of Medical Sciences and Peking Union Medical College Beijing China; ^2^ National Kunming High‐level Biosafety Primate Research Center, Institute of Medical Biology Chinese Academy of Medical Sciences and Peking Union Medical College Yunnan China

**Keywords:** brain, proteomic, SARS‐CoV‐2 Omicron strain, neurological consequences, rhesus macaques

## Abstract

The SARS‐CoV‐2 Omicron variant is more contagious than the original Alpha variant and can still cause neurological symptoms, including cognitive impairment. To gain a deeper understanding of the molecular mechanisms underlying these neurological effects, this study was conducted. Proteomic and phosphoproteomic analyses were carried out utilizing LC‐MS/MS. Samples included brainstem, cerebellum, frontal lobe, occipital lobe, parietal lobe, and temporal lobe from SARS‐CoV‐2 Omicron‐infected and noninfected control rhesus macaques. Infection with Omicron resulted in inflammatory responses across all six brain regions, and significant regional‐specific molecular alterations were observed. Proteomic and phosphoproteomic analyses revealed extensive abnormalities in immune activation, synaptic function, DNA repair, and signaling pathways across different brain regions. We further predicted key kinases and identified candidate proteins with significantly altered expression. This study reveals region‐specific inflammatory and signaling signatures in the brain following Omicron infection. The recognized kinases and proteins with abnormal regulation serve as potential candidates for drug discovery. This can aid in formulating new therapeutic approaches for neurological issues related to COVID‐19.

## Graphical abstract

The study found that SARS‐CoV‐2 Omicron infection causes region‐specific inflammatory, immune, synaptic, and signaling disorders in the brain, and the predicted kinases and differentially expressed proteins may serve as potential targets for COVID‐19‐related drug discovery.

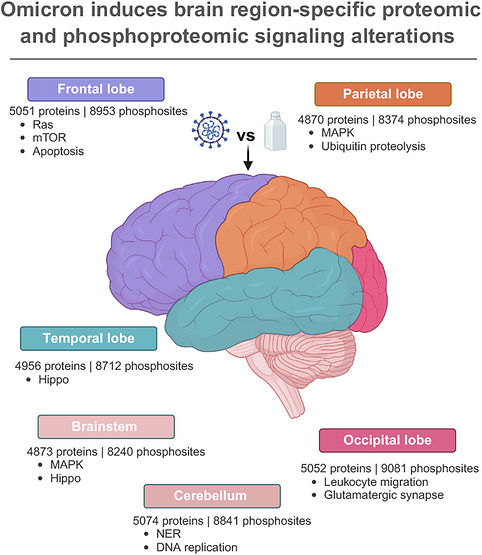

## 1 Introduction

As reported by the World Health Organization (WHO), the global health crisis instigated by the severe acute respiratory syndrome coronavirus 2 (SARS‐CoV‐2), commonly known as the COVID‐19 pandemic, had led to more than 779 million officially verified cases and 7 million deaths across the globe by May 24, 2026 [1]. The virus first came to light in December 2019. Since then, it has given rise to a succession of worrying variants. In 2020, variants such as alpha, beta, delta, and gamma emerged. In 2021, the Omicron variant appeared, accompanied by its sublineages BA.2, BA.4, and BA.5 that came later.

Notably, SARS‐CoV‐2 is not just a respiratory virus; it also shows clear neurotropism, which allows it to penetrate the central nervous system. The main ways it invades are by crossing the brain barriers or spreading through neural pathways [2]. The virus can pass through the blood–brain barrier using a transcellular method or access brain tissue via the blood‐cerebrospinal fluid barrier, especially through the choroid plexus epithelial cells that express the crucial receptors angiotensin‐converting enzyme 2 (ACE2) and transmembrane protease, serine 2 (TMPRSS2) [3, 4]. Moreover, the virus might be transmitted in a retrograde manner along peripheral nerves, like the olfactory nerve, through axonal transport. This mechanism is supported by animal models and certain autopsy studies [2]. On a molecular scale, apart from the main receptor ACE2, elements such as neuropilin‐1 (NRP1) could also facilitate the virus's entry into neurons and glial cells [5, 6]. Autopsy investigations have found viral RNA and proteins in the brain tissues of some deceased patients, and animal trials have verified viral replication and spread within the brain, demonstrating its direct neuroinvasive ability [7, 8]. Nevertheless, it is essential to stress that, when compared with the extensive respiratory infections, direct viral invasion of the central nervous system that leads to severe pathological alterations is still a relatively uncommon occurrence. Neurological symptoms are more likely to be indirectly driven by intense systemic immune and inflammatory responses. These direct neuroinvasive and indirect neuroinflammatory processes are considered critical pathological bases for the neurological clinical manifestations observed in patients [2].

Around thirty percent of COVID‐19 survivors show neurological symptoms, according to additional clinical research, even though the majority of COVID‐19 patients initially have breathing problems that can progress to serious lung conditions [9, 10, 11]. These can range from cognitive dysfunction and headaches to the lack of smell and taste, along with more severe issues like strokes, delirium, encephalitis, encephalopathy, primary psychiatric syndromes, and peripheral neuropathy [12, 13, 14]. Nonetheless, some neurological manifestations are short‐lived and mainly present themselves during the acute stage of severe COVID‐19. Others, like cognitive dysfunction, can linger for months on end, a condition known as Long COVID [15, 16, 17]. The quality of life and job opportunities for people impacted and those close to them can be seriously jeopardized by these persistent problems [18]. The risk landscape of neurologic manifestations was comparable before and following the Alpha variant's emergence, whereas the onset of the Delta variant correlated with heightened vulnerabilities to ischemic stroke, epileptic seizures, cognitive impairments, sleep disturbances, and anxiety disorders, coupled with a stark rise in mortality rates [19].

Following its emergence as the globally dominant strain, the Omicron variant has exhibited a distinctive epidemiological profile characterized by “high transmissibility and low acute lethality” [19]. Although it is associated with reduced acute disease severity and mortality compared with variants such as Delta [19, 20], structural biology studies have revealed that extensive mutations in its spike protein confer potent antibody evasion while maintaining efficient cellular infectivity [20]. This combination forms the fundamental basis for its unprecedented transmissibility [20]. Large‐scale epidemiological surveillance further confirms that Omicron's transmissibility far exceeds that of prior variants, leading to exponential growth in the number of infections [21]. However, crucial data from long‐term cohort studies indicate that this dramatic shift in transmission dynamics has not been accompanied by a reduction in the risk of neuropsychiatric sequelae; the relative risk of developing conditions such as Long COVID and cognitive impairment remains comparable to that observed during the Delta variant period [19]. This paradox of “high transmissibility coupled with persistent neurological risk” presents a unique public health challenge posed by Omicron: a variant seemingly milder in the acute phase may, due to its immense scale of infection, be generating an unprecedented and substantial burden of chronic neurological disease. Nonetheless, the underlying molecular mechanisms driving this clinical feature, particularly its specific perturbations to central nervous system signaling networks, remain largely unknown. Currently, there is no definitive cure for cognitive impairments and other neurological symptoms associated with the condition. As a result, it is of utmost necessity to conduct a more in‐depth exploration of the potential mechanisms that cause neurological outcomes after being infected with the SARS‐CoV‐2 Omicron variant. Moreover, it is essential to pinpoint practical therapeutic targets.

Phosphorylation stands as the most prevalent posttranslational alteration of proteins. It mainly takes place on serine residues, with threonine and tyrosine residues following in frequency. Given that kinases and phosphatases are responsible for catalyzing the phosphorylation and dephosphorylation processes, which, in turn, activate or deactivate numerous enzymes and receptors, this mechanism is a vital cellular regulatory process. Specifically, protein kinases play a key role in cellular signal transduction. The development and advancement of several neurological disorders, such as retinoblastoma, Parkinson's disease, Alzheimer's disease, and even COVID‐19, can be influenced by their dysregulation. This dysregulation might involve abnormal expression levels or changed catalytic activity of particular kinases, leading to abnormal phosphorylation of downstream substrates [22, 23, 24]. However, research on the brain tissues of COVID‐19 patients has largely concentrated on transcriptomics and proteomics [25, 26, 27, 28]. There is a distinct lack of studies regarding the phosphoproteome. The only relevant study was carried out on the cerebral cortex and hippocampus of mice [29]. In contrast to mice, rhesus macaques have more physiological and genetic similarities with humans. As a result, they are a more appropriate model for investigating the physiological mechanisms through which the SARS‐CoV‐2 Omicron strain impacts the nervous system and causes neurological effects [30].

Thus, we examined the proteome and phosphoproteome of the frontal lobe, occipital lobe, parietal lobe, temporal lobe, cerebellum, and brainstem in rhesus macaques infected with the SARS‐CoV‐2 Omicron strain and compared them with noninfected controls. This study generates an all‐encompassing dataset related to the proteomes and phosphoproteomes of multiple brain areas linked to COVID‐19. Moreover, it delves into the relationship between phosphorylation processes in the brain and the neurological impacts of SARS‐CoV‐2. We anticipate that this research will lay a theoretical groundwork and pinpoint potential therapeutic targets for addressing and preventing the neurological sequelae of SARS‐CoV‐2 infection.

## 2 Results

### 2.1 Proteomic and Phosphoproteomic Profiling of the Frontal, Occipital, Parietal, Temporal, Cerebellar Regions, and the Brainstem in Control Rhesus Macaques and Those Infected with the SARS‐CoV‐2 Omicron Strain

**FIGURE 1:**
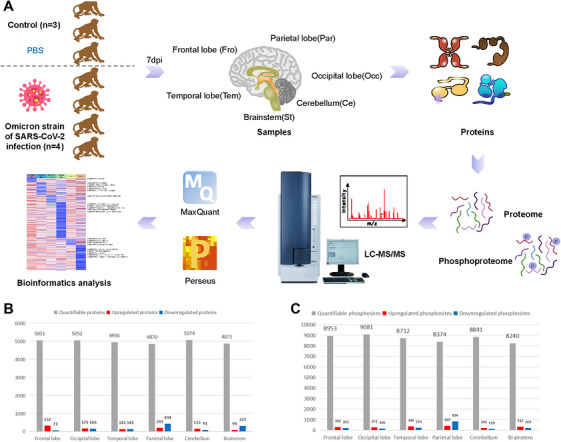
Global proteomic and phosphoproteomic profiling of the frontal lobe, occipital lobe, temporal lobe, parietal lobe, cerebellum, and brainstem in control (*n* = 3) and Omicron strain‐infected (*n* = 4) rhesus macaques. (A) Workflow for the rhesus macaques’ multiple brain regions proteome and phosphoproteome analyses. (B, C) Each brain region in the group infected with the Omicron strain (*n* = 4) was contrasted with its counterpart in the control group (*n* = 3). Quantifiable and differentially expressed proteins (fold change FC > 1.5 or < 0.67; *t*‐test significance, *p*‐value < 0.05) in each region are presented in (B). Quantifiable and differentially expressed phosphosites (FC > 1.5 or < 0.67; *t*‐test significance, *p*‐value < 0.05) in each region are presented in (C).

In this investigation, we analyzed six different brain regions in three mock‐infected and four rhesus macaques infected with the SARS‐CoV‐2 Omicron strain. The macaques were divided into a control group and an Omicron group. The macaques in the Omicron group were given an intranasal injection of the SARS‐CoV‐2 Omicron strain at a dose of 1 × 10^6^ tissue culture infective doses (TCID_50_). Meanwhile, the control group was administered an equal volume of PBS. Seven days postinfection, the rhesus macaques were humanely put to sleep. Subsequently, brain samples were carefully gathered from these animals. These samples were used for evaluating the viral load, conducting a detailed morphological inspection, and performing in‐depth proteomic and phosphoproteomic analyses using LC‐MS (Figure 1A; Table S1).

The results of the SARS‐CoV‐2 nucleic acid load test (Figure S1A) indicated that rhesus macaques infected with the virus exhibited similar infection intensities in different parts of the brain. However, no viral RNA was detected in certain brain areas in some individuals, while the brainstem and temporal lobe were consistently identified in all infected subjects. This variation might be attributed to differences in susceptibility to infection among the macaques or could result from experimental errors or other unforeseen factors. Post‐SARS‐CoV‐2 infection, we observed localized hemorrhaging, vasculitis, and the infiltration of inflammatory cells, along with a corresponding escalation in the pathological scoring of the affected brains (Figure S1B).

In the context of fundamental bioinformatics analysis, we carried out comparisons between the brain areas of the Omicron cohort and those of the control cohort. We used MaxQuant software to search the database, then Perseus software to screen for quantifiable proteins and phosphosites, and then used the Student's *t*‐test for difference analysis (fold change (FC) > 1.5 or < 0.67; *t*‐test significance, *p*‐value < 0.05). Global proteomic and phosphoproteomic profiling across six brain regions revealed widespread molecular dysregulation following Omicron infection. The number of quantified proteins and phosphosites, as well as the counts of significantly upregulated and downregulated molecules in each region, are comprehensively detailed in Figure 1B,C, and Tables S2 and S3.

We employed principal component analysis (PCA) and Venn diagrams to succinctly illustrate the commonalities and differences among several brain regions, and between infected and noninfected states. The outcomes of the PCA demonstrated that infections caused by the Omicron variant brought about extensive alterations in the proteomic and phosphoproteomic substances within the frontal, parietal, occipital, and temporal lobes, as well as the cerebellum and brainstem of rhesus macaques. Compared with the cerebral cortex, there was a significant disparity in both overall molecular levels and the types of molecules in the cerebellum and brainstem, with distinct organ‐specific characteristics evident. Additionally, the Venn diagrams revealed that each brain region possesses a unique set of proteins and phosphosites, suggesting that biological responses to the SARS‐CoV‐2 Omicron strain may vary across different brain regions (Figures S2 and S3).

### 2.2 Uniqueness and Similarity of Proteomic Functional Changes in the Frontal, Occipital, Parietal, and Temporal Lobes, as Well as the Cerebellum and Brainstem

**FIGURE 2:**
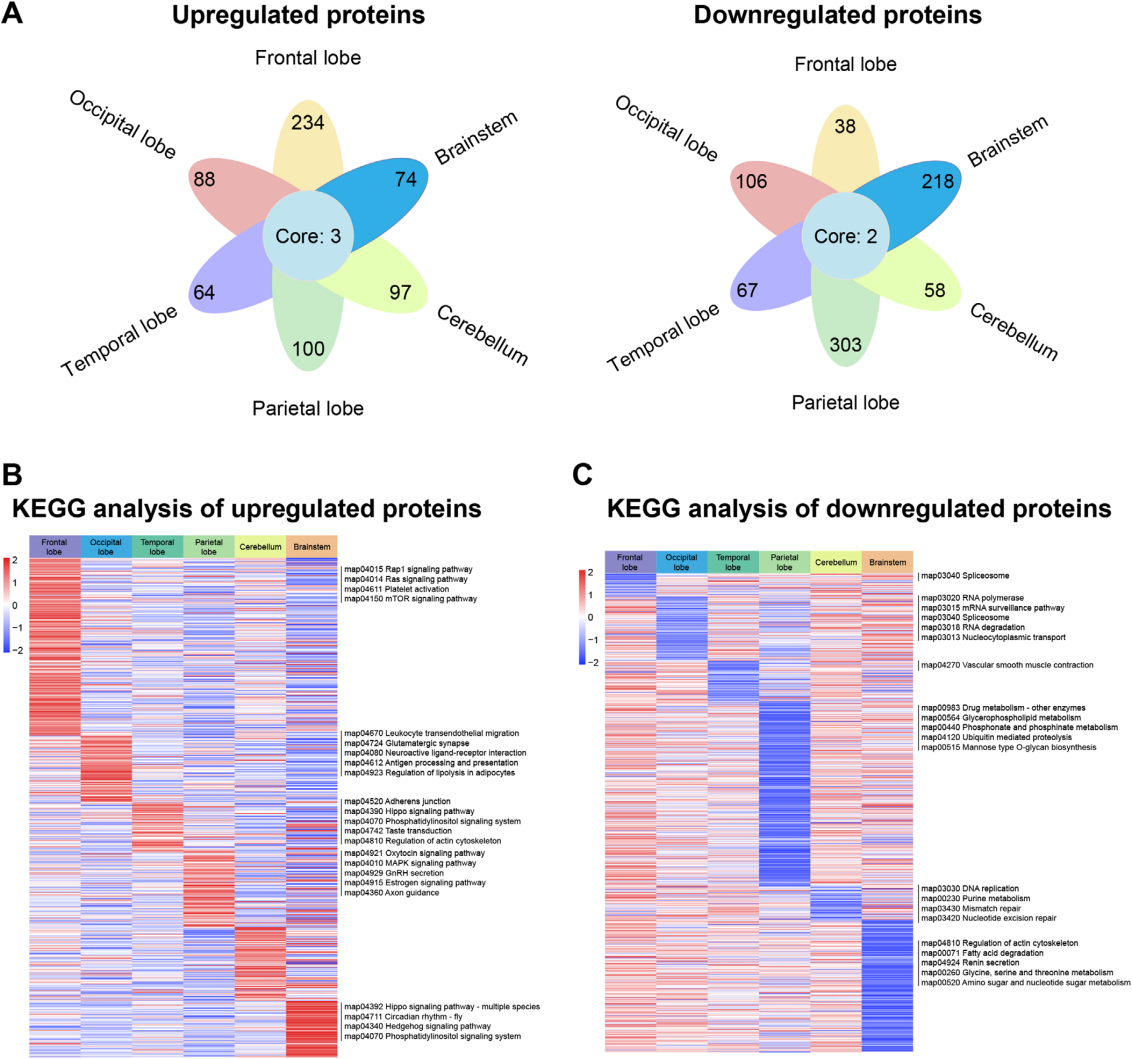
Bioinformatics analysis of differentially expressed proteins in multiple brain regions postinfection. (A) Petal plots of upregulated and downregulated proteins following infection in each brain region. The central portion of the plots represents proteins that are commonly upregulated or downregulated across all brain regions, whereas the outer sections highlight proteins that are uniquely altered in each specific brain region. (B) Heatmap of unique upregulated proteins in each brain region. Adjacent to the heatmap, a KEGG pathway enrichment analysis was conducted for these unique upregulated proteins, with the top five pathways for each region presented. (C) Heatmap of unique downregulated proteins in each brain region. Adjacent to the heatmap, a KEGG pathway enrichment analysis was conducted for these unique downregulated proteins, with the top five pathways for each region presented.

The proteins that are upregulated and downregulated across various brain regions are not identical. A petal chart was employed to depict the distinct upregulated and downregulated proteins within each specific brain region (Figure 2A). Subsequently, the Kyoto Encyclopedia of Genes and Genomes (KEGG) assessments were carried out on these proteins. After that, bioinformatics investigations were centered on the differentially expressed proteins that are specific to each brain region. We mapped the top five pathways with the most significant *p*‐values within each brain region (Figure 2B,C; Tables S4 and S5). Our findings indicate that the response to Omicron infection varies among different brain regions, and there is no single pathway common to all six regions.

**FIGURE 3:**
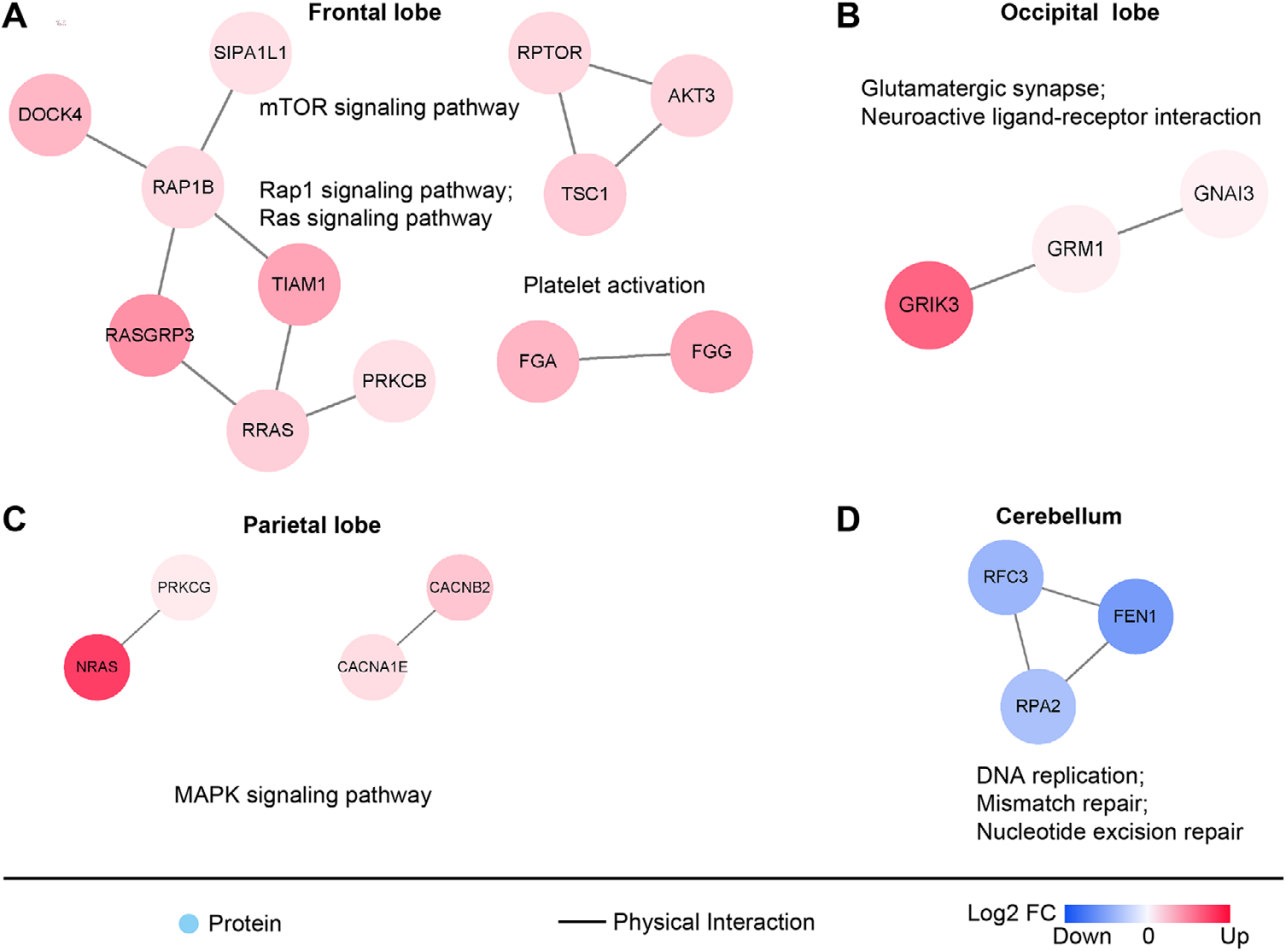
Protein–protein interaction (PPI) networks of proteins uniquely differentially expressed in four brain regions and enriched in major dysregulated KEGG pathways, with a confidence score ≥ 0.7. The red‐blue gradient color scheme indicates the log_2_ FC of the corresponding proteins within each specific brain region, providing a visual representation of the extent of upregulation or downregulation of the proteins involved in these pathways. (A) The PPI network of key KEGG pathways in the frontal lobe. (B) The PPI network of key KEGG pathways in the occipital lobe. (C) The PPI network of the key KEGG pathway in the parietal lobe. (D) The PPI network of key KEGG pathways in the cerebellum.

Our study identified a clear pattern following Omicron infection. The proteins that were upregulated were predominantly engaged in immune and inflammatory signaling cascades, which implies their possible functions in neurological harm, such as taste impairment. Downregulated proteins, by contrast, were largely associated with metabolic processes and protein homeostasis. To better understand the key mechanisms related to neural injury, we additionally built protein–protein interaction (PPI) networks for the major dysregulated pathways (Figure 3A–D).

Immune and inflammatory processes, like leukocyte transendothelial migration and antigen processing and presentation, were specifically enriched in the occipital lobe, indicating obvious neuroinflammation and immune cell infiltration in this region. Meanwhile, pathways closely related to neural function, including glutamatergic synapse and neuroactive ligand–receptor interaction, were also upregulated, suggesting disruptions in synaptic transmission and neural connections.

In the frontal lobe, upregulated proteins were concentrated in the Rap1, Ras, mTOR, and platelet activation pathways, reflecting abnormal vascular function, platelet aggregation, and cellular metabolic stress.

In the parietal lobe, upregulated proteins were significantly enriched in the MAPK signaling pathway, while downregulated proteins participated in ubiquitin‐mediated proteolysis, indicating strong inflammatory activation and disrupted protein balance.

In the cerebellum, no prominent pathways were identified among the upregulated proteins, but DNA replication, nucleotide excision repair, and mismatch repair were markedly downregulated, suggesting impaired DNA repair capacity and genome stability.

Additional pathway changes were found in the temporal lobe and brainstem. In the temporal lobe, upregulated proteins were enriched in Hippo signaling, phosphatidylinositol signaling, and actin cytoskeleton regulation, whereas downregulated proteins were related to vascular smooth muscle contraction, indicating abnormal cerebrovascular tone and cytoskeletal disorganization.

In the brainstem, upregulated proteins were enriched in the Hippo and Hedgehog signaling pathways, while downregulated proteins were associated with fatty acid degradation, indicating distinctive metabolic and developmental changes in this region.

Although abnormalities in the spliceosome, RNA processing, protein export, and nucleocytoplasmic transport were detected in multiple brain regions, these changes likely represent general cellular stress responses rather than core pathological mechanisms specific to SARS‐CoV‐2 infection.

Together, these results reveal distinct proteomic changes across different brain regions after Omicron infection, highlighting immune activation, synaptic dysfunction, disordered signaling, and impaired DNA repair as key events in the associated neurological injury.

### 2.3 Uniqueness and Similarity of Phosphoproteomic Functional Changes in the Frontal, Occipital, Parietal, and Temporal Lobes, as Well as the Cerebellum and Brainstem

**FIGURE 4:**
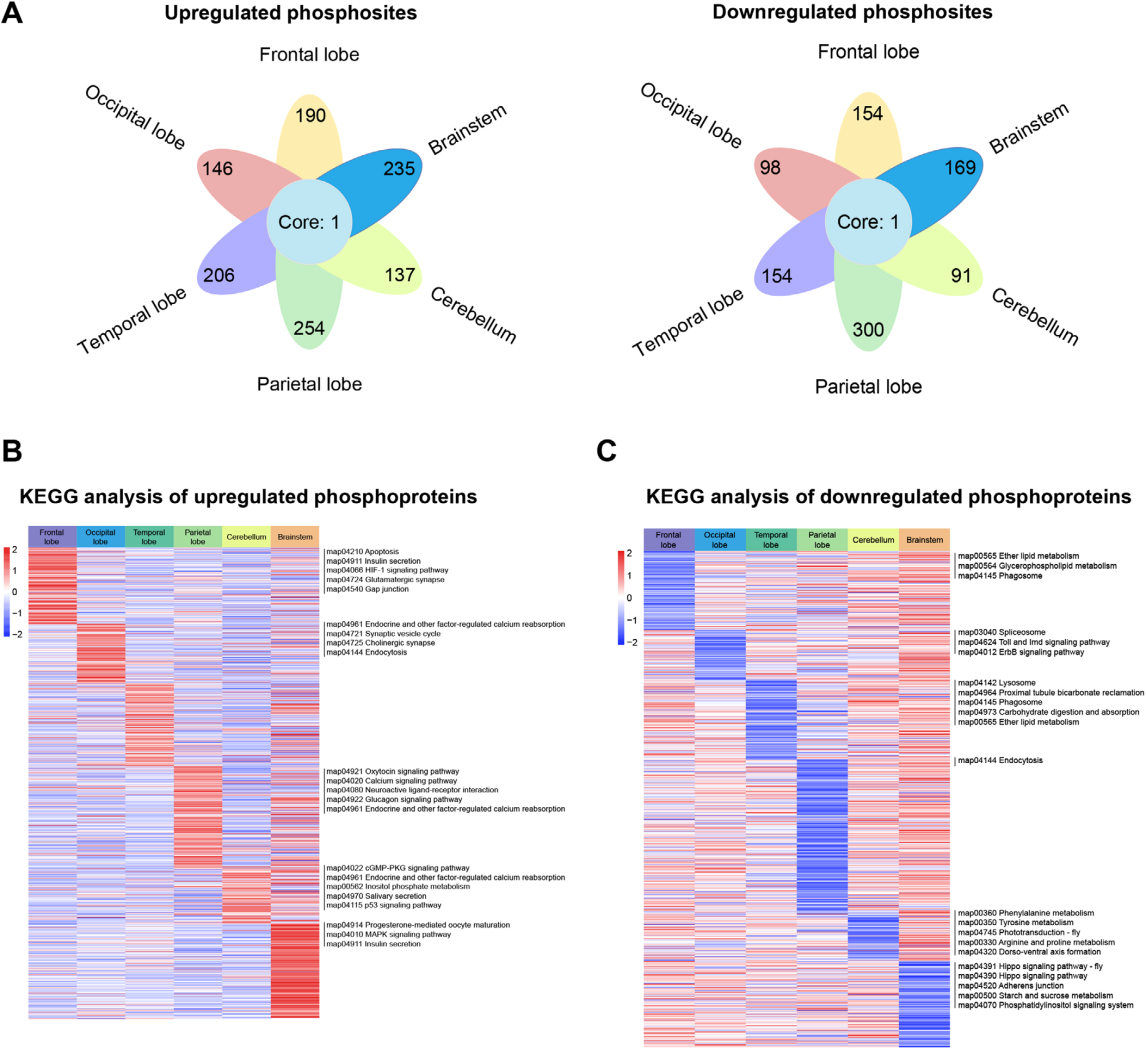
Bioinformatics analysis of differentially expressed phosphosites in multiple brain regions postinfection. (A) Petal plots of upregulated and downregulated phosphosites following infection in each brain region. The central portion of the plots represents phosphosites that are commonly upregulated or downregulated across all brain regions, whereas the outer sections highlight phosphosites that are uniquely altered in each specific brain region. (B) Heatmap of unique upregulated phosphosites in each brain region. Adjacent to the heatmap, a KEGG pathway enrichment analysis was conducted for these unique upregulated phosphosites, with the top five pathways for each region presented. (C) Heatmap of unique downregulated phosphosites in each brain region. Adjacent to the heatmap, a KEGG pathway enrichment analysis was conducted for these unique downregulated phosphosites, with the top five pathways for each region presented.

We employed a petal chart to vividly display the unique proteins, including those with upregulated and downregulated phosphosites, in each specific brain region (Figure 4A). These proteins were then examined using KEGG and PPI network analyses to elucidate their roles and interactions. We proceeded to map the top five pathways with the most significant *p*‐value within each brain region (Figure 4B,C; Table S6 and S7). Our findings reveal that the phosphoproteomic response to Omicron infection is distinct across various brain regions, with no single pathway being common to all six regions. This diversity underscores the heterogeneity of the different brain regions' reactions to the Omicron variant and highlights the complexity of its impact on the central nervous system.

Among these regions, proteins with upregulated phosphosites in the frontal lobe were enriched primarily in the HIF‐1 signaling pathway, apoptosis, and glutamatergic synapse pathways. These pathways are closely linked to neuronal survival and synaptic transmission, and constitute key events underlying functional disturbances in the frontal lobe. Proteins with downregulated phosphorylation, by contrast, were enriched in phagosome‐related pathways, implying altered immune clearance in this region.

In the occipital lobe, upregulated phosphoproteins were concentrated in endocytosis and synaptic vesicle cycling, both of which are central to viral entry and synaptic transmission. Conversely, downregulated phosphoproteins were associated with Toll‐like receptor signaling, pointing to distinct phosphorylation‐mediated changes in innate immune regulation.

In the parietal lobe, upregulated phosphoproteins were broadly enriched in calcium signaling and synapse‐related pathways, which govern ion homeostasis and neuronal signaling. Conversely, downregulated phosphoproteins were largely restricted to endocytosis, indicative of altered phosphorylation control over viral entry and synaptic function.

In the cerebellum and brainstem, upregulated phosphoproteins were enriched in the cGMP‐PKG pathway and p53‐related pathways, consistent with stress responses and cellular damage repair. Downregulated phosphoproteins were associated with cell junction organization and metabolic processes.

**FIGURE 5:**
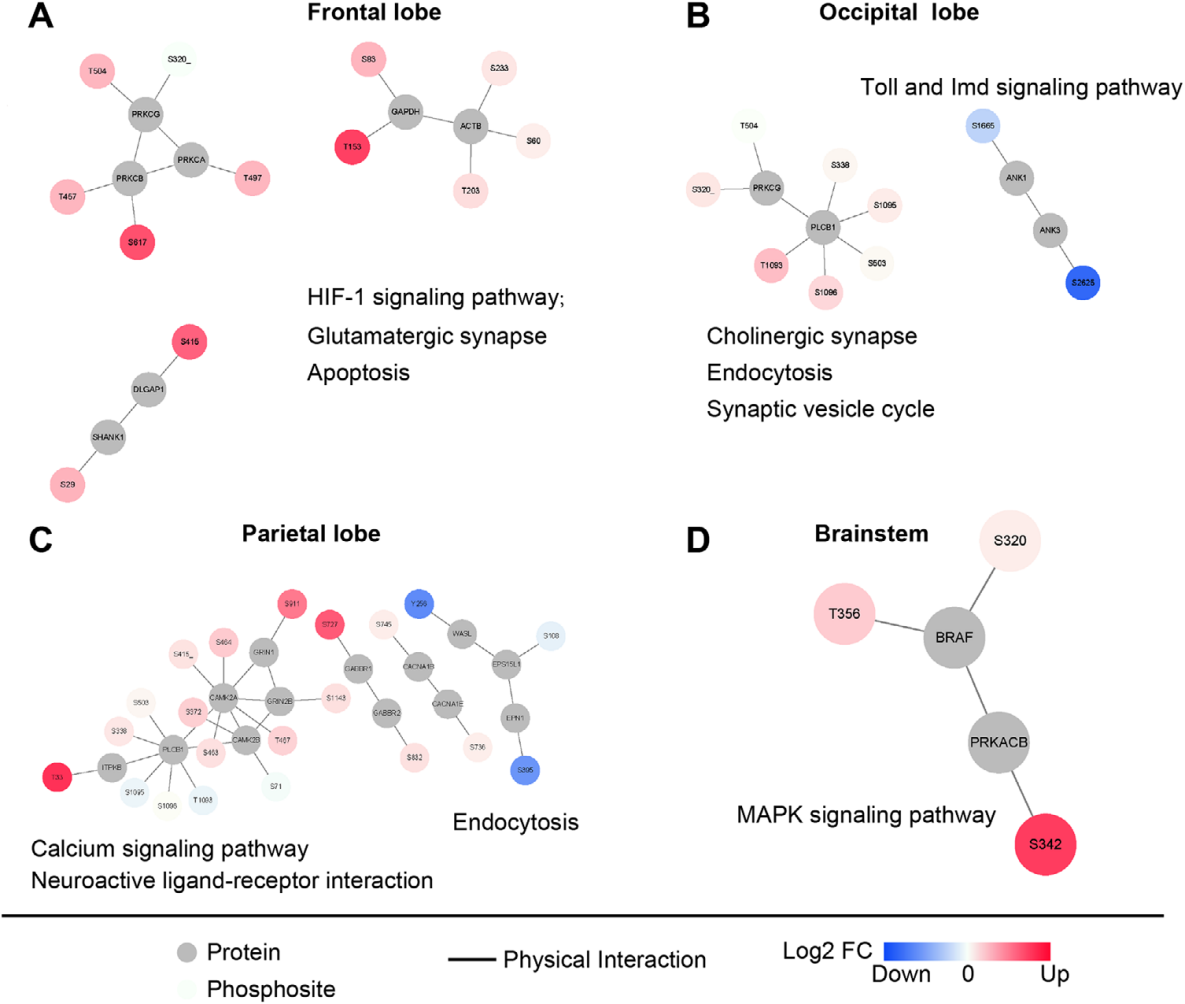
PPI networks for proteins with uniquely differentially expressed phosphosites in four brain regions and are enriched in the major dysregulated KEGG pathways (Figure 4B,C) with a confidence score ≥ 0.7. The red‐blue gradient color scheme indicates the log_2_ FC of the corresponding phosphosites within each specific brain region, providing a visual representation of the extent of upregulation or downregulation of the phosphosites involved in these pathways. (A) The PPI network of key KEGG pathways in the frontal lobe. (B) The PPI network of key KEGG pathways in the occipital lobe. (C) The PPI network of the key KEGG pathway in the parietal lobe. (D) The PPI network of key KEGG pathways in the brainstem.

Notably, the MAPK signaling pathway in the brainstem showed the most prominent upregulation of phosphorylation, representing a major cascade mediating local inflammation and neural injury. To better visualize the interactions among core proteins in these key pathways, we constructed region‐specific PPI networks (Figure 5A–D).

Overall, phosphorylation changes induced by Omicron infection were predominantly concentrated in pathways governing synaptic function, immune responses, and viral entry, with marked regional specificity across different brain regions. No single pathway was uniformly enriched across all regions, suggesting highly distinct responses to infection in different brain areas. Guided by these pathway‐level phosphorylation patterns, we next performed kinase prediction to identify key regulatory events underlying region‐specific neural damage following Omicron infection.

### 2.4 Kinase Prediction and Kinase Activity Analysis in each Brain Region

**FIGURE 6:**
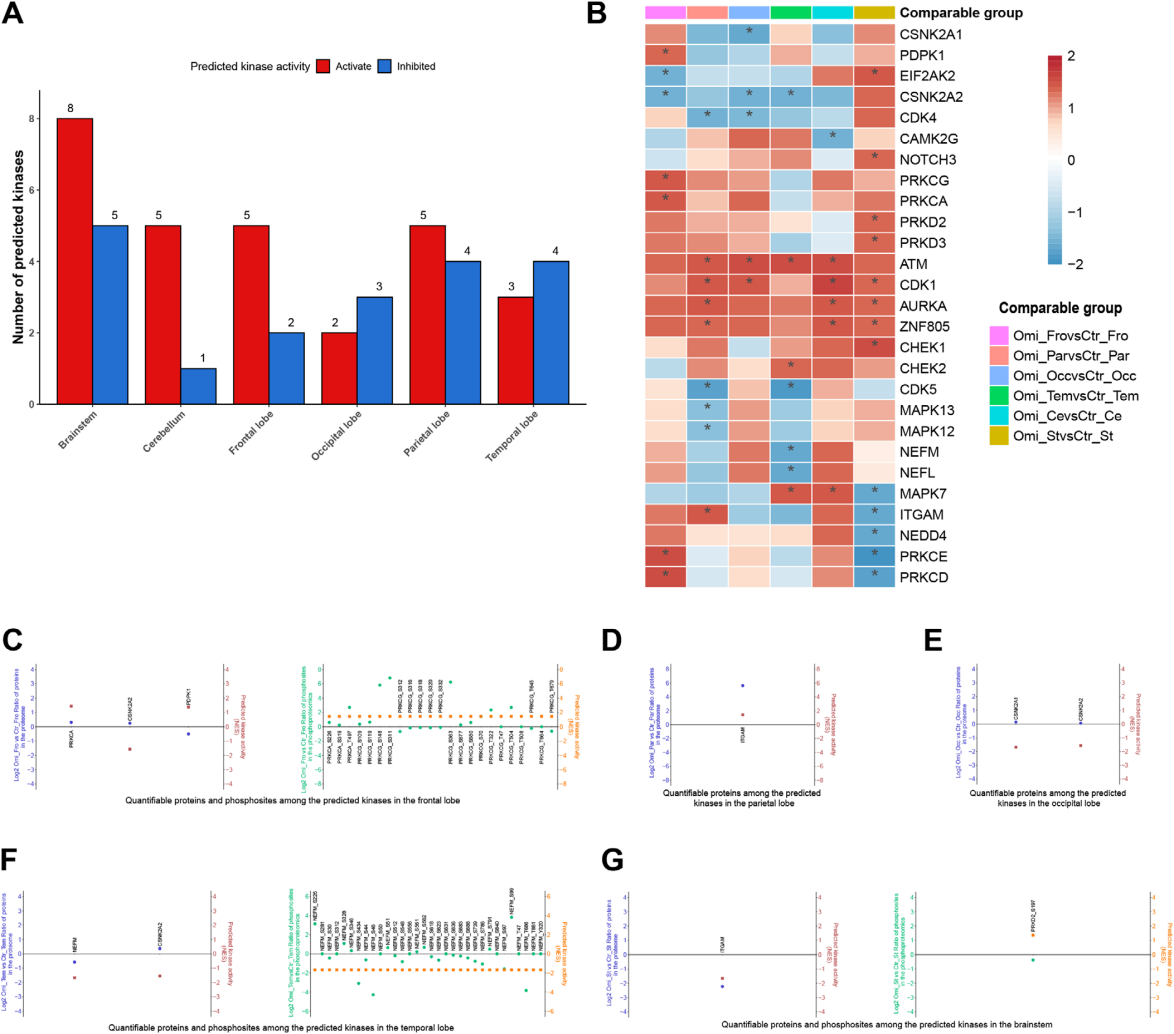
Phosphoproteome‐based kinase prediction and kinase activity prediction in each brain region. (A) The quantity of anticipated kinases in each brain region following SARS‐CoV‐2 Omicron strain infection. The Omicron‐infected group (*n* = 4) was compared with the control group (*n* = 3). Kinase prediction was conducted based on the quantification of phosphosites employing the GPS, and the GSEA was used to predict kinase activity. The NES value obtained from the GSEA indicates the expected kinase activity. The threshold for predicting activated kinases was set at NES > 1, *p*‐value <0.05, and the threshold for predicting inhibited kinases was set at NES < −1, *p*‐value <0.05. (B) Heatmap of predicted kinases with markedly changed activity following SARS‐CoV‐2 Omicron strain infection. NES values are used to represent kinase activity. * means NES > 1 or < −1, *p*‐value < 0.05. (C–G) Comparison of expected kinase activity and the actual identified FC in the frontal lobe (C), parietal lobe (D), occipital lobe (E), temporal lobe (F), and brainstem (G) after SARS‐CoV‐2 Omicron strain infection (*n* = 4, *t*‐test, *p < 0.05, **p < 0.01, ***p < 0.001). Each region of the brain has quantifiable proteins or phosphosites that correspond to the expected kinases. The proteome's quantifiable protein changes and the associated kinase activity are displayed in the left panel. The quantitative alterations of phosphosites in the phosphoproteome and the associated kinase activity are displayed in the right panel. Red and orange squares represent the NES of the kinases, and blue and green dots represent the log_2_ FC of proteins or phosphosites, respectively.

We submitted quantifiable phosphosites to the GPS to establish phosphorylation‐kinase relationships, predicting a total of 340 kinases (Table S8). Afterward, we utilized the GSEA algorithm to evaluate kinase activity. Kinases were considered to have significantly changed activity if they had a GSEA *p*‐value of less than 0.05 and a normalized enrichment score (NES) greater than 1 (indicating activation) or less than −1 (indicating inhibition). Our results showed that after infection with the SARS‐CoV‐2 Omicron strain, a number of kinases were activated in six different brain regions. Specifically, 5 kinases were activated in the frontal lobe, 2 in the occipital lobe, 3 in the temporal lobe, 5 in the parietal lobe, 8 in the brainstem, and 5 in the cerebellum. On the other hand, several kinases were inhibited in these six brain regions as well. There were 2 inhibited kinases in the frontal lobe, 3 in the occipital lobe, 4 in the temporal lobe, 4 in the parietal lobe, 5 in the brainstem, and 1 in the cerebellum (Figure 6A; Table S9). Notably, the total number of inhibited kinases was higher than that of activated kinases.

Furthermore, heatmap analysis has unveiled distinct activation patterns of specific kinases in various brain regions, each with unique implications for cellular processes: CDK1, a key regulator in cell division and proliferation, shows activation in the occipital, parietal, cerebellar, and brainstem regions. CDK1 is also associated with Parkinson's disease, Alzheimer's disease, stroke, and other neurological diseases [31]. This implies that CDK1 might be essential for the cellular response to infection in these areas. ATM, integral to DNA damage repair mechanisms [32], exhibits activation in the occipital, temporal, parietal, and cerebellar lobes. This indicates that DNA repair pathways may be actively engaged in response to infection in these regions. EIF2AK2, which is important to the immune system's reaction to viral infections, appears to be suppressed in the frontal lobe but is activated in the brainstem [33]. This dual behavior of EIF2AK2 highlights the complex and region‐specific nature of the immune response within the brain. Interestingly, no kinases were found to be consistently activated or inhibited across all six brain regions, indicating that the activity of kinases is uniquely patterned in each region following infection. This regional specificity of kinase activity underscores the nuanced and diverse responses of the brain to viral invasion (Figure 6B; Tables S2 and S3).

For validation, we compared all quantifiable proteins and phosphosites with the predicted activated or inhibited kinases. The FC and NES for kinases and their phosphosites are presented as dot plots (Figure 6C–G; Tables S2 and S3). Except for the cerebellum, other brain regions exhibited matching kinases and quantifiable proteins or phosphosites. We observed that the patterns for NES and FC were unstable, indicating that the expected activated or inhibited kinases may either upregulate or downregulate the associated proteins/phosphosites.

### 2.5 Interactions between the Viral and Host Proteins of the SARS‐CoV‐2 Omicron Strain were Studied in the Frontal, Occipital, Parietal, and Temporal Lobes, as well as the Cerebellum and Brainstem of Rhesus Macaques

**FIGURE 7:**
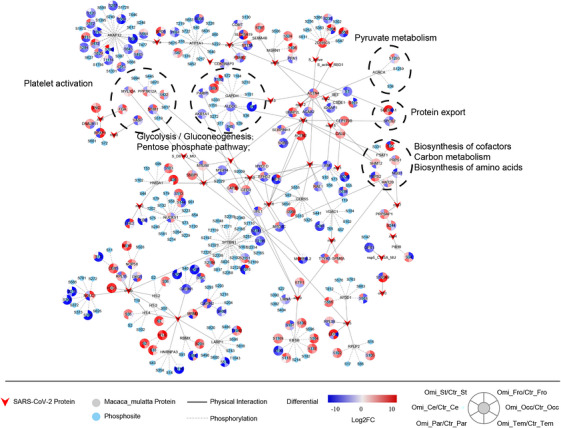
PPI networks of SARS‐CoV‐2 viral proteins, host differentially expressed proteins, and host differentially expressed phosphosites in the six brain regions of control and SARS‐CoV‐2 Omicron strain‐infected rhesus macaques. Leveraging the SARS‐CoV‐2 protein interactome from Yadi Zhou and colleagues, we have delineated these interaction networks. 30 SARS‐CoV‐2 proteins potentially interact with 93 host proteins that encompass 239 phosphosites. The host proteins were analyzed by the KEGG database to predict their functions, identifying 19 proteins enriched in eight KEGG pathways as illustrated. The SARS‐CoV‐2 Omicron strain's viral proteins are denoted by red arrows. Circles with gray centers represent host proteins, and those with blue centers signify host phosphosites. The surrounding gradient from red to blue indicates the extent of quantitative changes in proteins or phosphosites across brain regions postinfection.

SARS‐CoV‐2 Omicron strain encodes a set of proteins that are crucial for its replication and pathogenesis. There are four principal structural proteins, namely the membrane protein (M), envelope protein (E), spike protein (S), and nucleocapsid phosphorylated protein (N). Moreover, the virus generates 16 nonstructural proteins (nsps) and nine accessory proteins. These proteins perform diverse functions in regulating the host cell's mechanism [34]. Building on previous research into the interactions between SARS‐CoV‐2 and human proteins [35], we established a comprehensive network that delineates the interactions among SARS‐CoV‐2 proteins, rhesus macaque proteins, and phosphosites, as detailed in Figure 7 and Table S10. We utilized BLAST to align differentially expressed proteins and phosphosites in rhesus macaques with their human counterparts. Our analysis identified 30 SARS‐CoV‐2 proteins with the potential to interact with 93 host proteins, involving 239 phosphorylation sites. Notably, several of these proteins are implicated in the platelet activation pathway, pyruvate metabolism pathway, protein export pathway, and multiple glucose metabolic pathways.

The complex interaction between these viral proteins and their corresponding host proteins, especially at phosphorylation sites, implies that they could be viable candidates for therapeutic measures against the SARS‐CoV‐2 Omicron strain infection. By regulating these interactions, it might be possible to disrupt the virus's ability to commandeer biological functions, presenting a novel approach for the development of COVID‐19 treatments.

### 2.6 Validation of Proteomic Candidate Protein

To further validate the quantitative proteomic data, signal transducer and activator of transcription 1 (STAT1) was selected for Western blot analysis. Proteomic profiling revealed consistent upregulation of STAT1 across all examined brain regions of SARS‐CoV‐2‐infected rhesus macaques. In this study, we conducted additional protein‐level validation focusing on the parietal and temporal lobes. Consistent with the proteomic findings, STAT1 protein abundance was markedly elevated in the infected parietal and temporal lobe tissues (Figure S4).

## 3 Discussion and Conclusion

In the present study, we conducted an analysis of the proteomes and phosphoproteomes of diverse brain regions in both control rhesus macaques and those infected with the Omicron strain. The results show that infection with the Omicron variant can induce specific protein and phosphorylation signal‐remodeling in brain regions. Specifically, this manifests as excessive regional immune inflammation activation, synaptic transmission dysfunction, impaired DNA repair mechanisms, and disordered related signaling pathways. Moreover, the damage pattern is not uniform throughout the brain, suggesting a highly heterogeneous response of the central nervous system to SARS‐CoV‐2 infection. Previous multi‐omics investigations on COVID‐19 patients and studies using nonhuman primate models have likewise discovered that COVID‐19 infection can cause particular immune disruptions and synaptic malfunctions in various brain regions. The outcomes of these prior studies are highly congruent with the findings of our current research. Consequently, our study furnishes a comprehensive omics foundation for clarifying the molecular mechanisms underlying neuroinjury induced by the Omicron variant [36, 37]

In this study, SARS‐CoV‐2 RNA was consistently detectable in the brainstem and temporal lobe of infected animals. In contrast, positive results were only obtained in some individuals for other brain regions. At present, there are notable disparities in the detection of viral nucleic acids in the brain across different studies. These disparities may be strongly associated with factors such as sample origins, the time of sampling postinfection, detection sensitivity, individual immune reactions, and the varying inherent susceptibilities of different brain regions [7, 37, 38, 39, 40]. The stable detection of viral RNA in the brainstem and temporal lobe suggests that these regions are more susceptible to Omicron, while the molecular changes observed in brain regions without viral detection are more likely to be mediated by indirect pathways such as systemic inflammation and cytokine storm, indicating that both direct viral invasion and secondary neuroinflammation jointly participate in the occurrence and development of COVID‐19‐related neurological damage.

It is commonly known that people with the Omicron strain infection of COVID‐19 often develop taste disorders [41, 42]. The extensive neuroinflammation observed in this study and the disruption of synaptic transmission pathways may serve as an important molecular basis for postinfection sensory dysfunction. The related changes may involve signaling molecules such as PLCB4, which, as a core member of the phosphatidylinositol pathway, participates in intracellular signal transduction and is associated with various disease processes [43, 44]. Abnormal calcium signaling in the parietal lobe and phosphorylation of synaptic‐related pathways may also contribute to the occurrence of olfactory transmission disorders by disrupting ion homeostasis and synaptic transmission efficiency. Changes in the expression and modification of molecules such as CAMK2A, CAMK2B, and SLC8A2 may also play a certain role in this process [45].

There is substantial regional heterogeneity in the pathological responses to Omicron infection across distinct brain areas. This heterogeneity arises from inherent differences in regional function, cellular composition, and intrinsic susceptibility to viral and inflammatory injury. The frontal lobe, as the center of higher cognitive function, displays prominent disruptions in intracellular signaling and metabolic stress. The occipital and parietal lobes, which mediate sensory information processing, are characterized by marked synaptic dysfunction and localized neuroinflammation. The cerebellum exhibits marked vulnerability to DNA damage, as reflected by robust downregulation of DNA repair pathways. The brainstem, a critical region for autonomic and vital function, shows heightened viral susceptibility and stress‐related signaling and metabolic alterations. Collectively, these observed molecular patterns align well with the known structural and functional specializations of each brain region [36, 37, 38].

It is worth noting that after being infected with the SARS‐CoV‐2 Omicron variant, the expression of the phosphosite of α‐synuclein (SNCA) at Ser42 showed a significant increase in the frontal lobe and a significant decrease in the temporal lobe. SNCA, which is mainly expressed in the adult central nervous system and mostly located in the cytoplasm, is believed to play a role in regulating synaptic function and neuroplasticity [46]. Clustered SNCA proteins are cytotoxic, and highly phosphorylated, aberrantly aggregated SNCA serves as a pathogenic feature of Parkinson's disease and Lewy body dementia [47]. In a healthy brain, most SNCA exists in an unphosphorylated state. However, in the Lewy bodies of Parkinson's patients, more than 90% of the abnormally aggregated SNCA is phosphorylated, and this is regarded as having pathological implications [48, 49, 50]. There has been a documented rise in the incidence of illness and death among Parkinson's disease patients after SARS‐CoV‐2 infection [51]. Our findings could provide a theoretical basis for this phenomenon. Abnormal phosphorylation of SNCA indicates that Omicron infection can disrupt the stability of synaptic nuclear proteins, and over the long term, this may increase the risk of neurodegenerative changes. This is in line with the clinical symptoms of some COVID‐19 survivors who still suffer from neurological after‐effects [52].

Our research also specifically examined microtubule‐associated protein (MAPT). Neurofibrillary tangles (NFTs), which are composed of highly phosphorylated MAPT, are characteristic pathological features of primary tauopathies. These primary tauopathies include frontotemporal lobar degeneration with tau pathology (FTLD‐tau), progressive supranuclear palsy (PSP), and corticobasal ganglionic degeneration (CBD). Moreover, they act as pathological indicators for secondary tauopathies, such as Alzheimer's disease and chronic traumatic encephalopathy (CTE) [53, 54, 55, 56]. MAPT can undergo phosphorylation at numerous sites. However, when MAPT is phosphorylated, its affinity for microtubules typically decreases. This reduction in affinity causes MAPT to have a greater tendency to self‐aggregate, which in turn results in tau disease [57, 58]. In our investigation, after infection with the SARS‐CoV‐2 Omicron strain, not only was MAPT upregulated in the frontal, parietal, and occipital lobes, but the expression levels of its 14 phosphosites also changed across different brain regions. This finding implies that Omicron infection can undermine the stability of the neuronal cytoskeleton. This disruption may be linked to the decline in cognitive speed and inattention witnessed in some patients during the recovery phase. It indicates a possible connection between COVID‐19 infection and long‐term neurodegenerative risks [52, 59].

Although there is significant heterogeneity in the pathological changes of various brain regions, the downstream key genes of interferons—such as ISG15, IFIT1, and IFIT3—are significantly upregulated in all brain regions. This suggests that central antiviral immune activation mediated by type I interferons is a common event after Omicron infection. The continuous high expression of these molecules can amplify the neuroinflammatory response and have a negative impact on synaptic structure integrity and signal transmission function, thereby accelerating the process of nerve damage. The expression levels of HAPLN2 and VCAN are generally downregulated in multiple brain regions, indicating damage to the white matter structure and extracellular matrix homeostasis. HAPLN2 is crucial for maintaining myelin integrity and nerve conduction velocity, and its downregulation may lead to slowed nerve conduction, which is an important structural basis for fatigue, cognitive sluggishness, and other sequelae in long‐term COVID patients; the downregulation of VCAN further weakens the inflammatory regulation and tissue repair ability, and both of them jointly promote the continuous progression of nerve damage [60, 61, 62].

To sum up, this study offers thorough proteome and phosphoproteome information on the brainstem, cerebellum, frontal lobe, occipital lobe, parietal lobe, and temporal lobe of rhesus macaques following infection with the SARS‐CoV‐2 Omicron strain. Excessive activation of immune inflammation, disordered synaptic transmission function, decreased DNA repair capacity, and abnormal brain region‐specific signaling pathways jointly constitute the core molecular mechanism of neuroinjury related to Omicron. The regional distribution of viral RNA in the brain and the differences among studies can be explained by the timing of sample collection, detection methods, and brain region susceptibility. Both direct viral invasion and systemic inflammatory responses jointly mediate central pathological damage. This study provides multilevel omics evidence for understanding the mechanisms of acute neurological complications and long‐term neurological sequelae related to Omicron, and lays a data foundation for the screening and mechanism verification of potential intervention targets in the future.

### 3.1 Limitations of the Study

Several limitations of the present study warrant acknowledgment. First, brain regions from rhesus macaques were utilized due to the insufficient availability of brain tissues from COVID‐19 patients. Although rhesus macaques exhibit greater homology to humans compared with conventional experimental animals, interspecies differences remain, which may affect the reliability of the findings. Importantly, the ages of the infected animals did not correspond to those of the uninfected controls, raising the possibility that some observed molecular differences reflect developmental stage rather than viral infection. Consequently, interpretations regarding potential age‐related effects should be approached with caution. Additionally, this study was limited to bioinformatics analyses and did not include in‐depth experimental validation of the key proteins identified. To address this critical limitation, forthcoming research will prioritize experimental confirmation, including (i) verification of expression levels and posttranslational modifications of pivotal targets (e.g., MAPT, SNCA, and other key proteins identified with observed changes) in tissue lysates via Western blot; and (ii) development of in vitro cellular models, such as SARS‐CoV‐2‐infected neuronal or glial cells, to conduct functional gain‐and loss‐of‐function assays assessing the influence of these targets on viral replication and inflammatory responses. These planned investigations are essential to substantiate the biological roles of the identified targets in COVID‐19‐associated neurological sequelae and will be detailed in subsequent studies.

## 4 Materials and Methods

### 4.1 Virus Culture, Amplification, and Titration

The SARS‐CoV‐2 Omicron variant (B.1.1.529) was procured from the Institute of Laboratory Animal Science at the Chinese Academy of Medical Sciences. The National Kunming High‐level Biosafety Laboratory received authorization from the National Health Commission for the import and transportation of this particular virus strain. Following this approval, the virus was amplified and stored within the laboratory facility. SARS‐CoV‐2 demonstrates high adaptability in Vero E6 cells [63]. The process of virus harvesting began by inoculating the virus into twenty T225 cell culture flasks. Once distinct cytopathic effects (CPE) were observable under a microscope, the virus was collected roughly 72 h later and then stored at a temperature of −80°C. The next day, the stored virus was gradually thawed at 37°C. After thawing, it was centrifuged, and the supernatant was then put through an ultrafiltration process to concentrate the virus. The concentrated virus was then washed three times using phosphate‐buffered saline (PBS). To obtain a concentrated virus solution, the washed virus was eluted with 200 mL of PBS. Finally, a plaque assay was carried out to measure the viral titer [64].

### 4.2 Animal Experiments

In this research, seven rhesus macaques (*Macaca mulatta*) were employed. This group consisted of four rhesus macaques infected with the SARS‐CoV‐2 Omicron strain (within the Omicron‐infected cohort: four 2‐year‐old females) and three uninfected control macaques (in the control group: two females and one male, aged 8 to 11 years). All these animals were sourced from the Institute of Medical Biology, Chinese Academy of Medical Sciences. After anesthesia, the Omicron strain (titer of 1×10^6^ PFU/mL) was administered to the rhesus macaques in the Omicron strain‐infected group via a combination of intranasal droplet administration with 500 µL and intratracheal injection with 500 µL, resulting in a total inoculum dose of 1 × 10^6^ PFU per monkey. The control group was administered an equivalent volume of PBS. The body temperature and body mass of the rhesus macaques infected with the Omicron strain were continuously tracked. Seven days after inoculation (7 dpi), the animals were humanely killed before undergoing a necropsy. Subsequently, tissue specimens from the frontal lobe, occipital lobe, temporal lobe, parietal lobe, cerebellum, and brainstem were collected from both the control and the Omicron‐infected groups. These samples were used for detecting the viral load, conducting histopathological examinations, and performing proteomic and phosphoproteomic analyses. After that, the samples were stored at a temperature of −80°C.

In the control group, considering ethical requirements and the scarcity of rhesus macaques, we used the minimum sample size of three animals, as determined by an a priori statistical power calculation. To ensure the robust establishment of the infection model, four rhesus macaques were included in the Omicron infection experiment. Fortunately, all of them successfully established the infection model. Consequently, to guarantee the creation of a strong infection model while complying with the ethical and practical limitations regarding the utilization of limited nonhuman primates, we carried out sampling and subsequent examinations on three rhesus macaques in the control cohort and four in the group infected with the Omicron variant.

### 4.3 Bioinformatics Analysis

All bioinformatics assessments were carried out utilizing the differentially expressed proteins and differentially regulated phosphosites specified in the “Quantification and Statistical Analysis” segment (Supporting Information Materials and Methods) (*p*‐value less than 0.05 and FC greater than 1.5 or less than 0.67).

A KEGG pathway enrichment analysis was carried out by means of KOBAS (version 3.0, available at http://kobas.cbi.pku.edu.cn/) and Python (version 3.9, based on Fisher's exact test). Statistical significance was denoted by a *p*‐value lower than 0.05. The STRING website, specifically version 12.0 (https://string‐db.org/), was employed to generate protein–protein interaction (PPI) networks. A confidence score of 0.7 was used, which was considered the highest level of confidence. Subsequently, the interaction networks of proteins were visualized with the help of Cytoscape version 3.8.2 (https://cytoscape.org/download.html) [65]. We made use of the BioLadder platform (https://www.bioladder.cn/web/#/pro/index) to build principal component analysis (PCA) diagrams. Venn diagrams were produced using the R package VennDiagram (version 1.7.3). Petal plots and heatmaps were crafted using R (version 4.3.1), with the pheatmap package (version 1.0.12) being used for creating heatmaps. The graphical abstract was generated with BioRender (https://biorender.com). To convert a protein from one species into an analogous protein from another, we made use of NCBI's BLAST version 2.13.0.

The prediction of kinases was carried out by quantifying phosphosites with the use of GPS version 5.0 (https://gps.biocuckoo.cn/download.php) [66]. Additionally, GSEA was carried out to forecast kinase activity (version 4.0.3, https://www.gsea‐msigdb.org/gsea/index.jsp) [67]. The anticipated kinase activity is represented by the NES value generated by the GSEA algorithm. An NES greater than 1 and a *p*‐value less than 0.05 served as the criterion for predicting activated kinases. Conversely, an NES less than −1 and a *p*‐value less than 0.05 was the standard for predicting inhibited kinases.

## Author Contributions

Qiaochu Wang, Yanan Zhou, Tao Ding, and Yan Sun share the first authorship. Juntao Yang and Jiangfeng Liu: Conceptualization, resources, formal analysis, writing – review and editing, supervision, project administration. Shuaiyao Lu, Yan Sun, and Yanan Zhou: Methodology, formal analysis. Qiaochu Wang: Methodology, software, formal analysis, visualization, writing – original draft, writing – review and editing. Tao Ding: Formal analysis, writing – review and editing. Yehong Yang and Yue Wu: Formal analysis. All authors read and approved the final manuscript.

## Funding

This research was funded through funding from the Prevention and Control of Emerging and Major Infectious Diseases‐National Science and Technology Major Project (2025ZD01900200), the Chinese Academy of Medical Sciences Innovation Fund for Medical Sciences (2025‐I2M‐KJ‐013, CIFMS2022‐I2M‐1‐011 and CIFMS2022‐I2M‐2‐001), the National Key R&D Program of China (2023YFC2507102), and State Key Laboratory Special Fund 2060204.

## Ethics Statement and Consent to Participate

The animal experiments were reviewed and approved by the Laboratory Animal Ethics Committee of the Institute of Medical Biology, Chinese Academy of Medical Sciences, with the approval number: DWSP202203034. Throughout the evaluation process, all animal experiments were conducted strictly by biosafety operation standards and ethical requirements for animal welfare, ensuring humane care and the well‐being of the animals. Experimental procedures were carried out under anesthesia to meet both biosafety requirements and ethical guidelines. All animal experiments were performed at the National Kunming High‐level Biosafety Primate Research Center in Kunming, Yunnan, China, following the “National Guidelines for Animal Care and Use” recognized by the national authorities for animal research.

## Conflicts of Interest

The authors declare no conflicts of interest.

## Supporting information




**Supporting File 1**: mco270906‐sup‐0001‐SuppMat.pdf

Supporting File 2: mco270906‐sup‐0002‐Tables.xlsx

## Data Availability

The dataset supporting the conclusions of this article is publicly available in the ProteomeXchange repository, PXD056745 (submitted via iProX) [68, 69]. This includes all raw mass spectrometry files, database search results, and processed quantitative datasets, as well as statistically analyzed data supporting downstream biological analyses.
