# Supplementary material for: Proteomic and Phosphoproteomic Characteristics of Multiple Brain Regions in Rhesus Macaques After SARS‐CoV‐2 Infection
Source: MedComm (2020). 2026 Aug 6;7(8):e70906. doi: 10.1002/mco2.70906 (PMC13448139; doi:10.1002/mco2.70906)
Supplement: Supplementary file 1 [file MCO2-7-e70906-s002.pdf]

Supplemental information

Proteomic and phosphoproteomic characteristics of multiple brain regions in rhesus macaques after SARS-CoV-2 infection

Running title: Multiple brain regions' proteomes

Journal name: MedComm

Qiaochu Wang[1,#], Yanan Zhou[2,#], Tao Ding[1,#], Yan Sun[1,#],Yehong Yang[1], Yue Wu[1], Jiangfeng Liu[1, *], Shuaiyao Lu[2, *] , Juntao Yang[1, *]

---

[1] *State Key Laboratory of Common Mechanism Research for Major Diseases, Department of Biochemistry and Molecular Biology, Institute of Basic Medical Sciences, Chinese Academy of Medical Sciences & Peking Union Medical College, Beijing 100005, China*

[2] *National Kunming High-level Biosafety Primate Research Center, Institute of Medical Biology, Chinese Academy of Medical Sciences and Peking Union Medical College, Yunnan 650118, China.*

[#] **These authors contributed equally.**

[*] **Corresponding author information:**

Jiangfeng Liu: ljf@pumc.edu.cn (01065105980)

Shuaiyao Lu: lushuaiyao-km@163.com (087168335402).

Juntao Yang: yangjt@pumc.edu.cn (01065105980).

Juntao Yang is the paper's lead contact.

Supplementary figures

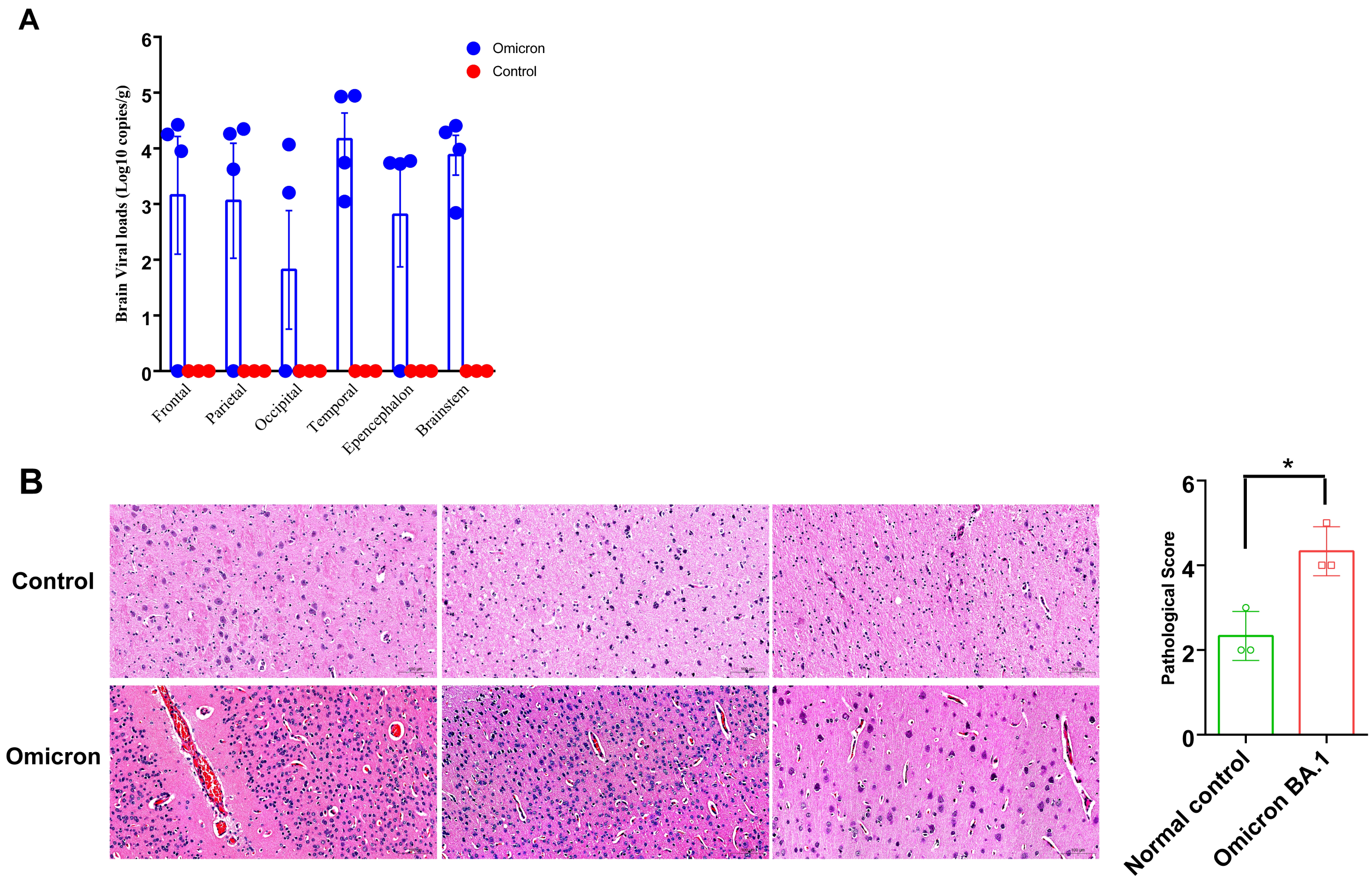

Figure S1. Viral load detection and H&E staining of the frontal lobe, occipital lobe, temporal lobe, parietal lobe, cerebellum, and brainstem in control and SARS-CoV-2 Omicron strain-infected rhesus macaques.

A. SARS-CoV-2 Omicron strain viral loads in the frontal lobe, occipital lobe, temporal lobe, parietal lobe, cerebellum, and brainstem of control (n = 3) and SARS-CoV-2 Omicron strain-infected (n = 4) rhesus macaques. All data are represented as the mean ± SEM.

B. H&E staining of brain sections from control and SARS-CoV-2 Omicron strain-infected rhesus macaques.

The left panel displays brain sections. In the control group, the structure appears clear and intact, with regularly arranged cells, and no evident histopathological changes were observed. In contrast, the infected group exhibited increased local bleeding and inflammatory cell infiltration.

The right panel illustrates the pathological scores of the brain sections, with the Omicron strain-infected group (n=3) showing higher scores than the control group (n=3). All data are represented as the mean ± SEM, and unpaired t-test was used for statistical analysis. *$p < 0.05$.

The magnification for each image is 400x.

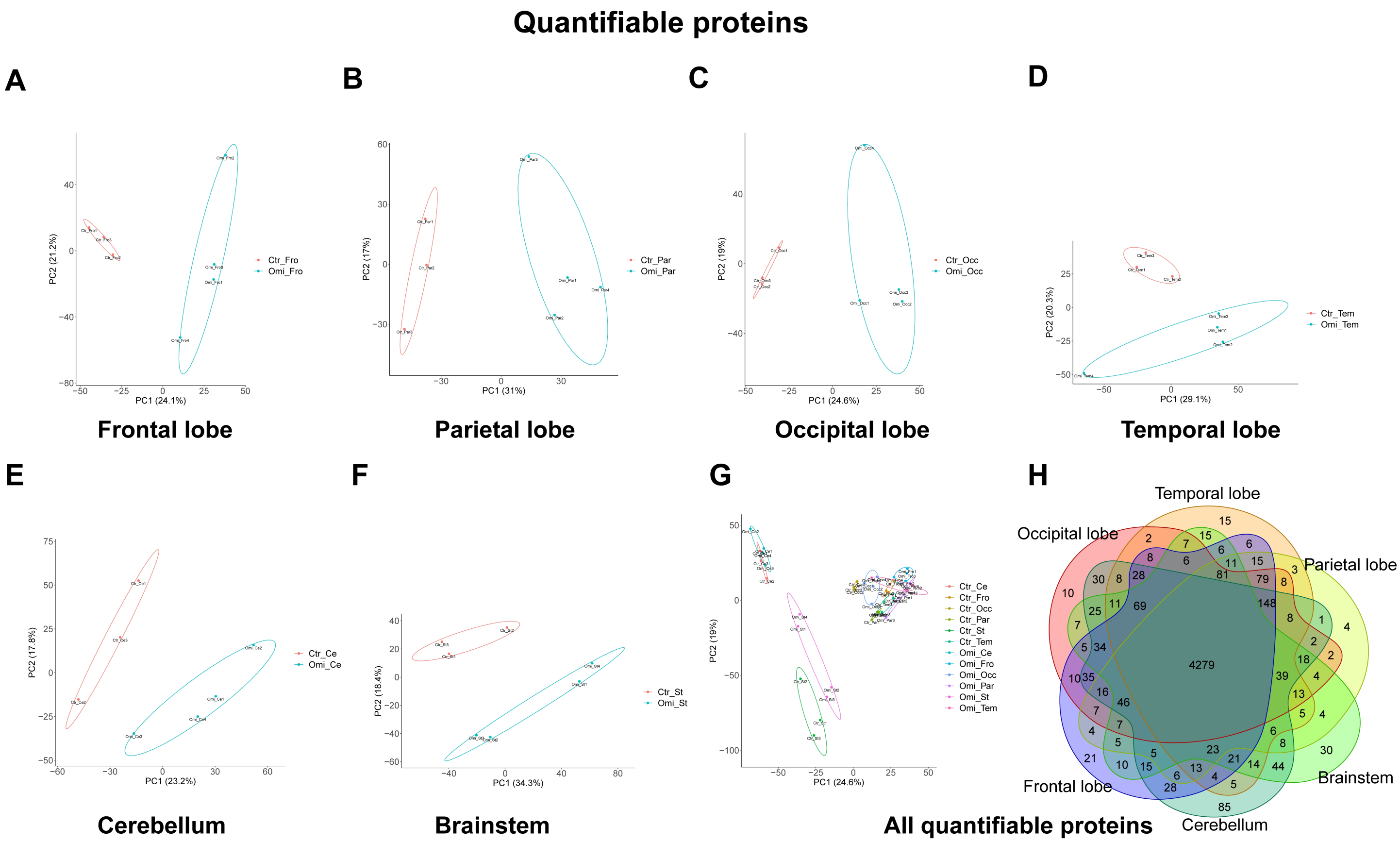

Figure S2. General characteristics of the label-free proteome determined using LC-MS/MS.

A − F. Principal component analysis (PCA) of quantifiable proteins in the proteome of the frontal lobe **(A)**, parietal lobe **(B)**, occipital lobe **(C)**, temporal lobe **(D)**, cerebellum **(E)**, and brainstem **(F)**.

G. PCA of all 5,484 intersecting quantifiable proteins across the six brain regions of control and SARS-CoV-2 Omicron strain-infected rhesus macaques.

H. Venn diagram of quantifiable proteins in the six brain regions of control and SARS-CoV-2 Omicron strain-infected rhesus macaques.

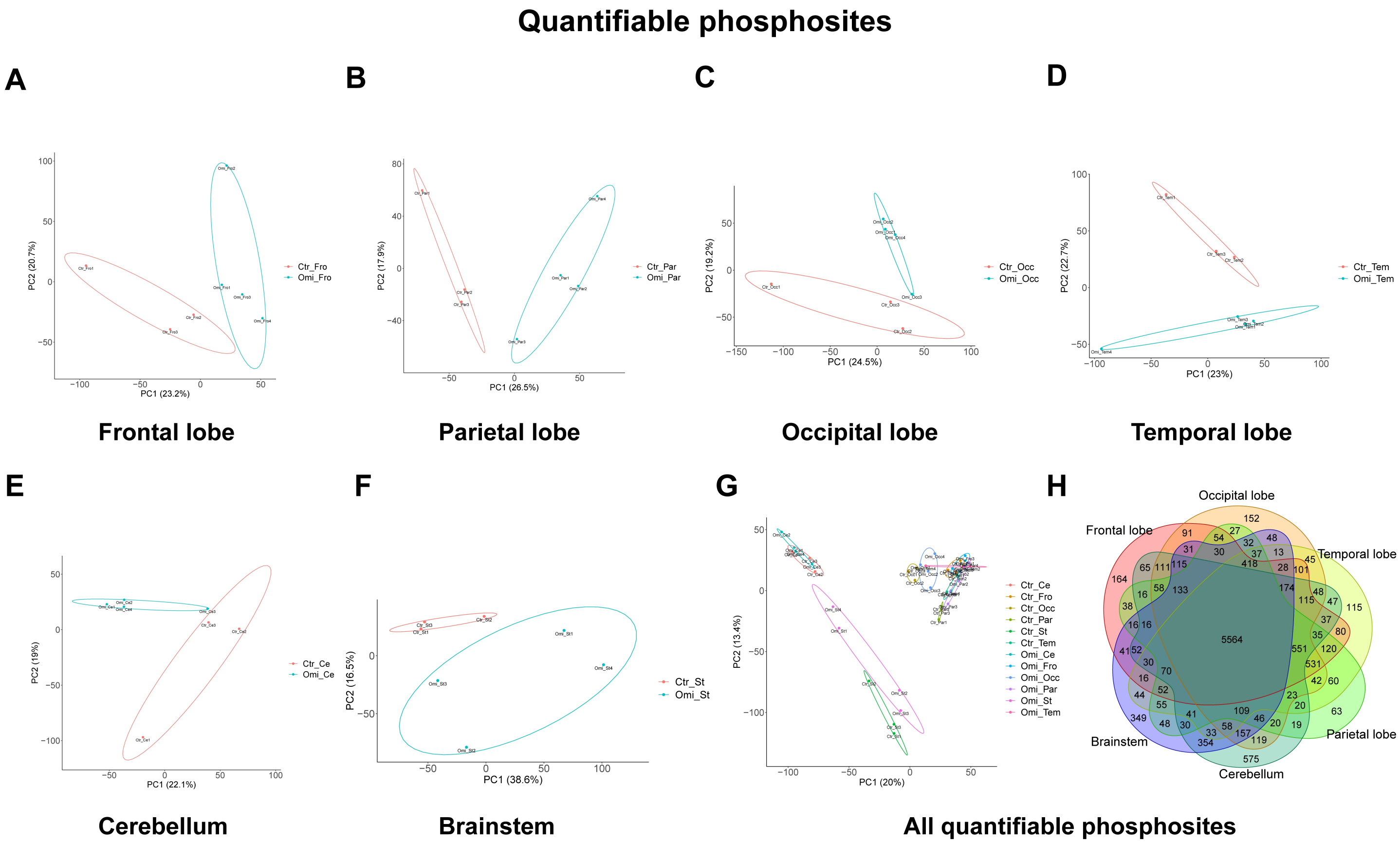

Figure S3. General characteristics of the label-free phosphoproteome determined using LC-MS/MS.

A – F. Principal component analysis (PCA) of quantifiable phosphosites in the phosphoproteome of the frontal lobe **(A)**, parietal lobe **(B)**, occipital lobe **(C)**, temporal lobe **(D)**, cerebellum **(E)**, and brainstem **(F)**.

G. PCA of all 11,782 intersecting quantifiable phosphosites across the six brain regions of control and SARS-CoV-2 Omicron strain-infected rhesus macaques.

H. Venn diagram of quantifiable phosphosites in the six brain regions of control and SARS-CoV-2 Omicron strain-infected rhesus macaques.

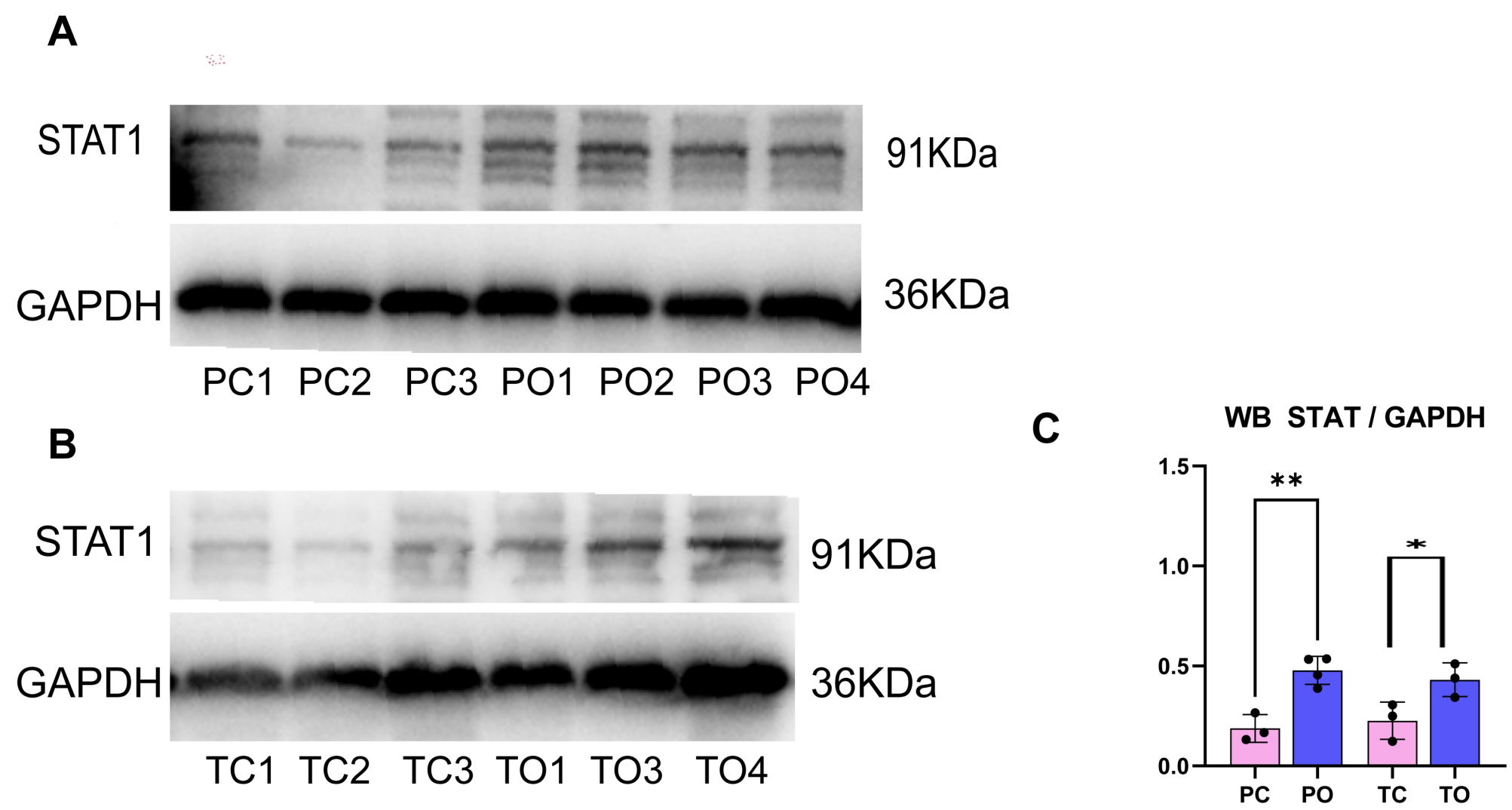

Figure S4. Representative Western blot images of STAT1 expression in the parietal and temporal lobes. A total of 14 samples were originally planned for analysis; however, one sample TO2, the second sample from the temporal lobe in the Omicron infection group, failed protein extraction, leaving 13 samples included in the final Western blot experiment.

A. Western blot bands showing STAT1 and internal reference GAPDH expression in the parietal lobe.

B. Western blot bands showing STAT1 and internal reference GAPDH expression in the temporal lobe.

C. Quantitative analysis of STAT1 band intensities normalized to GAPDH, analyzed using ImageJ and plotted in GraphPad Prism. PC, n=3; PO, n=4; TC, n=3; TO, n=3 . All data are represented as the mean ± SEM, and Welch's t test was used for statistical analysis (*$P < 0.05$, **$P < 0.01$).

Supplementary tables

Table S1. Animal information and viral loads of rhesus macaques.

Table S2. The identified quantifiable proteins in the frontal lobe, occipital lobe, parietal lobe, temporal lobe, cerebellum, and brainstem of rhesus macaques.

Table S3. The identified quantifiable phosphosites in the frontal lobe, occipital lobe, parietal lobe,

temporal lobe, cerebellum, and brainstem of rhesus macaques.

Table S4. KEGG pathway enrichment analysis of upregulated proteins in the frontal lobe, occipital lobe, parietal lobe, temporal lobe, cerebellum, and brainstem of rhesus macaques.

Table S5. KEGG pathway enrichment analysis of downregulated proteins in the frontal lobe, occipital lobe, parietal lobe, temporal lobe, cerebellum, and brainstem of rhesus macaques.

Table S6. KEGG pathway enrichment analysis of proteins containing upregulated phosphosites in the frontal lobe, occipital lobe, parietal lobe, temporal lobe, cerebellum, and brainstem of rhesus macaques.

Table S7. KEGG pathway enrichment analysis of proteins containing downregulated phosphosites in the frontal lobe, occipital lobe, parietal lobe, temporal lobe, cerebellum, and brainstem of rhesus macaques.

Table S8. Predicted kinases in the frontal lobe, occipital lobe, parietal lobe, temporal lobe, cerebellum, and brainstem of rhesus macaques.

Table S9. Predicted kinase activity in the frontal lobe, occipital lobe, parietal lobe, temporal lobe, cerebellum, and brainstem of rhesus macaques.

Table S10. SARS-CoV-2 Omicron strain-host protein interactomes identified in previous studies and the present study.

Supplementary Materials and methods

1. Plaque Assay

The infectious titer of the concentrated SARS-CoV-2 Omicron variant stock was ascertained via a

standard plaque assay employing Vero E6 cells. To begin, 5×10^5 Vero E6 cells were seeded per well in 12-well plates to form confluent monolayers, which were then incubated overnight. Subsequently, the viral stock was subjected to 10-fold serial dilutions. For each dilution, 200 µL was added in duplicate to the wells. The plates were then placed at 37 ℃ with 5% CO_2 for a 1-hour adsorption period. During this time, the plates were gently rocked every 15 minutes. After the adsorption period, the inoculum was removed, and 2 mL of maintenance medium was added to each well as an overlay. The plates were then incubated at 37 ℃ with 5% CO_2 for 72 hours. Once the incubation was complete, the overlay medium was removed. The cells were then fixed and stained using 1 mL of crystal violet solution per well (Biological Industries, Israel). Following rinsing and air-drying, plaques were counted manually. Only wells containing between 30 and 300 plaques were included in the analysis to ensure statistical reliability. Viral titer (PFU/mL) was calculated using the following formula: Titer = (Average plaque count× Dilution factor) / 0.2 mL. The titer of the concentrated viral stock was determined to be 1×10^7 PFU/mL, based on consistent results obtained from independent replicate assays[1].

2. Morphological Analysis

To ensure proper fixation, samples of brain tissue were collected and immersed in 10% neutral-buffered formalin for a period ranging from three to seven days. Once the fixation process was completed, the tissues underwent processing and were embedded in paraffin. For the purpose of histological examination and evaluation, sections of the paraffin-embedded tissues, with a thickness of 5- μ m, were prepared and subsequently stained using hematoxylin and eosin (H&E)[2].

3. Extraction and Quantification of Viral RNA

In accordance with the manufacturer's guidelines, RNA was isolated from animal swab specimens or

tissue samples that were suspended in TRIzol reagent. The isolation process utilized the Direct-zol RNA Miniprep kit (Zymo Research, product number R2052). For swab samples, those immersed in TRIzol were vigorously vortexed, after which the swabs were taken out. A 400-microliter volume of the TRIzol suspension from the swab samples was employed for RNA extraction. Subsequently, 50 microliters of DNase/RNase -free water was used to elute the RNA, which was then stored at a temperature of -80 ℃.When it came to tissue sampling during dissection, around 100 milligrams of animal tissue was measured and homogenized in 800 microliters of TRIzol. The homogenized mixture was then centrifuged to extract the RNA template. After that, 50 microliters of DNase/RNase- free water was used to elute the RNA template. The prepared RNA template was stored at - 80 ℃ for later use.To quantify the viral genome, real - time reverse transcription-polymerase chain reaction (RT-qPCR) was carried out. The TaqMan Fast Virus 1-Step Master Mix (Thermo Fisher Scientific) was used, with SARS-CoV-2 RNA serving as the standard curve. The RT-qPCR was performed on the CFX384 Touch Real-Time PCR Detection System (Bio-Rad). The primer and probe sequences were sourced from the N gene. The forward primer sequence was 5'-GACCCCAAAATCAGCGAAAT-3', the reverse primer sequence was 5'-TCTGGTTACTGCCAGCCAGTTGAATCTG-3', and the probe sequence was 5'-FAM-ACCCGCATTACGTTTGGTGGACCMRA -3'. These sequences were based on the ones recommended by the WHO and the Chinese Center for Disease Control and Prevention (China CDC).The conditions for the RT-qPCR were as follows: an initial incubation at 25 ℃ for 120 seconds, followed by 15 minutes at 50 ℃, and then 120 seconds at 95 ℃. This was followed by 40 cycles, each consisting of 5 seconds at 95 ℃ and 30 seconds at 60 ℃.

4. Protein Extraction

Samples of brain tissue, including those from the frontal, occipital, temporal, and parietal lobes, as well as the cerebellum and brainstem, were obtained from both control rhesus macaques and those infected with the SARS-CoV-2 Omicron strain. These samples were retrieved from storage at -80 ℃, weighed, and then placed into a mortar that had been pre-cooled with liquid nitrogen. Subsequently, the samples were meticulously ground into a fine powder with the use of liquid nitrogen. Next, an appropriate quantity of lysis buffer was added to the samples of each group. The lysis buffer contained 1% sodium dodecyl sulfate (SDS), 1% protease inhibitor cocktail, and 1% phosphatase inhibitor cocktail. After homogenizing the mixture, it was subjected to centrifugation at 4 ℃ with a force of 12,000 × g for 10 minutes. Following centrifugation, the supernatant was carefully transferred to a new centrifuge tube. The protein concentration in the supernatant was then determined using a BCA protein assay kit.

5. Trypsin Digestion

In each sample, an equal quantity of enzymes was employed to digest the protein. First, lysis buffer was utilized to standardize the volume of the samples. Subsequently, a single volume of chilled acetone was introduced. Once the mixture was thoroughly mixed by vortexing, four volumes of chilled acetone were added. The liquid was then placed in an incubator at -20 ℃ for two hours to induce protein precipitation. Afterward, the samples were centrifuged at 4500 × g for five minutes at 4 ℃. The supernatant was then discarded, and the protein pellets were washed two or three times with chilled acetone to remove any residual buffer. The pellets were left to air-dry before being resuspended in a solution with a final concentration of 200 mM TEAB.

Following the addition of 5 millimolar (mM) of dithiothreitol (DTT), the specimens were subjected to a 30-minute incubation at 56 ℃ to disrupt the disulfide linkages. Subsequently, iodoacetamide

(IAM) was introduced to achieve a final concentration of 11 mM. The samples were then incubated for 15 minutes at ambient temperature in the absence of light to alkylate the reduced cysteine residues. Next, trypsin was incorporated at a ratio of 1:50 (trypsin:protein, mass- to-mass). The pellets were then evenly distributed by applying sonication to the samples. After this step, the samples were allowed to undergo an overnight enzymatic digestion at 37 ℃.

6. HPLC Classification

In the case of phosphoproteomic samples, fractionation was not carried out. Conversely, for proteomic samples, peptides were fractionated via high-pH reverse-phase high-performance liquid chromatography (HPLC) with an Agilent 300 Extend C18 column. This column had a particle size of 5 micrometers, an inner diameter of 4.6 millimeters, and a length of 250 millimeters. The fractionation process unfolds as follows: Over a period of 60 minutes, peptides were separated into 60 distinct fractions. This separation was achieved by using a gradient of acetonitrile ranging from 8% to 32% at a pH of 9. After the separation, the resulting fractions were combined into three groups. After merging, the fractions were lyophilized using vacuum freeze-drying for further processing.

7. Affinity Enrichment

Proteomics analysis did not require affinity enrichment; only phosphoproteomic analysis did. The peptides were first dissolved in an enrichment buffer composed of 50% acetonitrile and 0.5% acetic acid. Subsequently, the supernatant was transferred to pre-washed IMAC (Immobilized Metal Affinity Chromatography) material. This IMAC material was then placed on a rotating shaker and incubated under gentle agitation. Once the incubation was completed, the material underwent a

washing process. It was washed three times, first with a buffer solution containing 50% acetonitrile and 0.5% acetic acid, and then with a solution of 30% acetonitrile and 0.1% trifluoroacetic acid. After the washing steps, phosphopeptides were eluted using 10% ammonia water. The eluate was collected and then lyophilized through vacuum freeze-drying. After the drying process, desalting was carried out following the C18 ZipTip protocol. Finally, the sample was again vacuum freeze-dried and made ready for liquid chromatography - mass spectrometry (LC-MS) analysis.

8. LC-MS/MS Analysis

Proteomic Analysis: Peptides were solubilized in solvent A of the liquid chromatography mobile phase and segregated using an EASY-nLC 1200 ultra-high-pressure liquid chromatography (UHPLC) apparatus. Mobile phase A was an aqueous mixture containing 0.1% formic acid and 2% acetonitrile, whereas mobile phase B was an aqueous solution with 0.1% formic acid and 90% acetonitrile. The HPLC gradient was configured as follows: from 0 to 40 minutes, the proportion of B increased from 7% to 23%; from 40 to 52 minutes, it rose from 23% to 35%; from 52 to 56 minutes, it climbed from 35% to 80%; and from 56 to 60 minutes, it was kept at 80%. The flow rate was maintained at 500 nl/min.After being separated by the UHPLC system, the peptides were fed into the NSI ion source for ionization and then entered the Orbitrap Exploris mass spectrometer for assessment. The ion source voltage was set at 2300 V. Both the precursor ions and their fragment ions were detected and analyzed by means of a high-resolution Orbitrap. The full scan range for the first mass spectrometry (MS1) was set between 400 and 1200 m/z with a resolution of 60,000. The fixed first mass for the second mass spectrometry (MS2) was set at 110 m/z, with a resolution of 15,000, and TurboTMT was deactivated.Data collection was carried out using a data-dependent

acquisition (DDA) program. In this program, after the initial scan, the 25 most intense precursor ions were successively chosen for collision- induced dissociation (HCD) with 27% collision energy, followed by sequential MS2 analysis. To enhance the efficient use of the mass spectrometer, the automatic gain control (AGC) was set to 100%, the signal threshold was set to 50,000 ions/s, the maximum injection time was set to automatic, and the dynamic exclusion time for tandem mass spectrometry scans was set to 20 seconds to avoid redundant scanning of precursor ions.

Phosphoproteomic Analysis: The peptides were solubilized in solvent A of the liquid chromatography mobile phase and segregated using an EASY-nLC 1200 ultra-high-performance liquid chromatography (UHPLC) apparatus. Mobile phase A was an aqueous mixture containing 0.1% formic acid and 2% acetonitrile, whereas mobile phase B was an aqueous mixture containing 0.1% formic acid and 90% acetonitrile. The HPLC gradient was configured as follows: from 0 to 70 minutes, it increased from 3% to 20% of B; from 70 to 82 minutes, it rose from 20% to 30% of B; from 82 to 86 minutes, it climbed from 30% to 80% of B; and from 86 to 90 minutes, it was maintained at 80% of B, with a flow rate kept at 500 nl/min. After being separated by the UHPLC system, the peptides were fed into the NSI ion source for ionization and subsequently analyzed using an Orbitrap Exploris 480 mass spectrometer. The ion source voltage was set at 2200 V. Both the parent ions and their fragments were detected and analyzed via a high- resolution Orbitrap. The full scan scope for the first mass spectrometry (MS1) was set between 350 and 1600 m/z with a resolution of 60,000. The fixed first mass for the second mass spectrometry (MS2) was set at 110 m/z, with a resolution of 30,000, and TurboTMT was deactivated.Data collection was carried out using a data-dependent acquisition (DDA) program. After the initial scan, the 15 most intense

parent ions were chosen for higher - energy collisional dissociation (HCD) with a 27% collision energy, followed by successive MS2 analysis. To boost the efficient utilization rate of the mass spectrometer, the automatic gain control (AGC) was set to 100%, the signal threshold was set to 50,000 ions/s, the maximum injection time was set to 100 ms, and the dynamic exclusion time for tandem mass spectrometry scans was set to 30 seconds to avoid repeated scanning of parent ions.

9. Database Search

A database search application was employed to bring in the raw mass spectrometry data, and the configurations were set up in line with the experimental procedure for analysis. MaxQuant (version 1.6.15.0) was utilized to conduct a search on the MS/MS data for this particular experiment. The search parameters were configured as follows: The default setting of the database was Macaca_mulatta_9544_PR_20230529.fasta, which contained 44,389 sequences. A decoy database was added to assess the false discovery rate (FDR) arising from random matches. Moreover, a database of common contaminants was integrated to eliminate the impact of contaminating proteins on the identification outcomes. Trypsin/P was employed for enzymatic digestion. The number of permitted missed cleavage sites was set at 2. The minimum length of a peptide was set to 7 amino acid residues, and the maximum number of modifications per peptide was restricted to 5. The mass tolerance for the precursor ions in the First search and Main search was set to 20 ppm and 4.5 ppm respectively, and the mass tolerance for the fragment ions in the MS/MS was set to 20 ppm.

In proteomic samples, the carbamidomethylation of cysteine (Carbamidomethyl, C) was designated as a constant modification. Meanwhile, the variable modifications encompass the oxidation of methionine and the acetylation of the protein's N-termini. The FDR for both protein and

peptide-spectrum match (PSM) identification was established at 1%.

In the case of phosphoproteomic specimens, the C is likewise designated as a fixed modification. Meanwhile, variable modifications consist of the oxidation of methionine, the acetylation of protein N-termini, and the phosphorylation of serine, threonine, and tyrosine residues. Similarly, the FDR for protein and PSM identification is set at 1%[3-6].

10. Quantification and Statistical Analysis

In order to investigate how the host's cerebral regions respond to an infection by the SARS-CoV-2 Omicron variant, we procured samples from the frontal, occipital, parietal, and temporal lobes, as well as the cerebellum and brainstem, of four rhesus macaques that had been infected and three that remained uninfected. These samples were then subjected to proteomic and phosphoproteomic analyses.

The data obtained from MaxQuant were fed into Perseus version 1.6.5.0[7] for subsequent processing. Initially, proteins that corresponded to the MaxQuant - derived data and were potentially contaminants or part of the reverse database were removed. Only phosphosites with a localization probability exceeding 0.75 and proteins having more than 1 unique peptide (Class I) were retained. A protein or phosphosite was considered quantifiable when at least half of the samples in either the control or Omicron groups contained them. In order to normalize the quantifiable proteins and phosphosites in each sample, we divided their intensity by the corresponding median. Then, we used the lowest of the remaining values to fill in the global missing values. These retained proteins and sites were then utilized for subsequent bioinformatics studies, such as the Kyoto Encyclopedia of Genes and Genomes (KEGG) analysis. To categorize the differentially expressed proteins and

sites, we employed the Student's t test with a p value threshold of 0.05. A protein or phosphosite was regarded as upregulated if its p value was 0.05 and its fold change (FC) was greater than 1.5. Conversely, a protein or site was considered downregulated when its p value was 0.05 and its FC was less than 0.67.

11. Western Blot

After determining protein concentrations using a BCA protein assay kit, proteins were separated by 10% SDS-PAGE (Yeasen Biotechnology Co., Ltd., catalog number PG222) and transferred to PVDF membranes (Millipore Corporation). The membranes were then blocked with Western blocking solution (Beyotime, catalog number P0252). Subsequently, the membranes were incubated overnight at 4° C with primary antibodies against STAT1 (Cell Signaling Technology, catalog number 9172S, dilution 1:4000) and GAPDH (Abcam, catalog number ab8245, dilution 1:4000). After washing with TBST, the membranes were incubated for 1 hour with horseradish peroxidase-conjugated anti-rabbit (Abcam, catalog number ab6721, dilution 1:10,000) or anti-mouse secondary IgG (Abcam, catalog number ab205719, dilution 1:10,000). Protein bands were visualized using enhanced chemiluminescence. Images were developed using the ECL chemiluminescence method, and band intensities were analyzed with ImageJ software. Protein expression levels were normalized to GAPDH.

References

1 Yahalom-Ronen, Y., Tamir, H., Melamed, S. *et al.* A single dose of recombinant VSV-ΔG-spike vaccine provides protection against SARS-CoV-2 challenge. *Nat Commun.* 2020; 11, 6402.

2 Tang, X., Zhou, Y., Wu, J. *et al.* Proteomic and phosphoproteomic profiling of SARS-CoV-2-associated liver injury: a report based on rhesus macaques. *MedComm (2020).* 2023; 4, e358.

3 Tyanova, S., Temu, T. & Cox, J. The MaxQuant computational platform for mass spectrometry-based shotgun proteomics. *Nat Protoc.* 2016; 11, 2301-2319.

4 Liu, J. F., Wu, S. F., Liu, S. *et al.* Global Lysine Crotonylation Profiling of Mouse Liver. *Proteomics.* 2020; 20, e2000049.

5 Zhao, L., Cong, X., Zhai, L. *et al.* Comparative evaluation of label-free quantification strategies. *J Proteomics.* 2020; 215, 103669.

6 Humphrey, S. J., Karayel, O., James, D. E. *et al.* High-throughput and high-sensitivity phosphoproteomics with the EasyPhos platform. *Nat Protoc.* 2018; 13, 1897-1916.

7 Tyanova, S., Temu, T., Sinitcyn, P. *et al.* The Perseus computational platform for comprehensive analysis of (prote)omics data. *Nat Methods.* 2016; 13, 731-740.
